# Cerebrospinal fluid and blood levels of neurofilament light chain in Parkinson disease: A protocol for systematic review and meta-analysis

**DOI:** 10.1097/MD.0000000000021458

**Published:** 2020-07-31

**Authors:** HongZhou Wang, WanHua Wang, HaiCun Shi, LiJian Han, PingLei Pan

**Affiliations:** a Department of Neurology, Kunshan Hospital, Affiliated to Jiangsu University, Kunshan; b Department of Neurology; c Department of Central Laboratory, The Yancheng School of Clinical Medicine of Nanjing Medical University, Yancheng, PR China.

**Keywords:** blood, cerebrospinal fluid, meta-analysis, neurofilament light chain, Parkinson disease

## Abstract

**Background:**

Parkinson disease (PD) is a common neurodegenerative disorder. Elevations of neurofilament light chain (NfL) concentrations in the cerebrospinal fluid (CSF) and blood are a marker of neuronal/axonal injury and degeneration. However, CSF and blood NfL alterations in patients with PD from existing studies remain inconclusive. To better understand these conflicting data, we will conduct a meta-analysis.

**Methods:**

We will comprehensively search PubMed, Embase, and Web of Science databases from each database's inception to 7th June, 2020. This protocol will conform to the Preferred Reporting Items for Systematic review and Meta-Analysis Protocols. We will only include original studies published in English that evaluated differences of NfL concentrations in the CSF or blood between idiopathic PD patients and healthy controls. The Newcastle-Ottawa Scale will be used to evaluate the quality of the included studies. Meta-analyses will be carried out using the STATA software version 13.0. Between-group difference of NfL concentrations in the CSF and blood will be expressed as the weighted standardized mean difference. A random-effects model will be used. Supplementary analyses, such as heterogeneity analysis, sensitivity analysis, publication bias, subgroup analysis, and meta-regression analysis will be performed.

**Results:**

The meta-analysis will provide the differences of NfL concentrations in the CSF and blood between patients with PD and healthy controls and will show the magnitudes of their effect sizes.

**Conclusions:**

This meta-analysis will provide the evidence of NfL concentrations in the CSF and blood in PD and we hope that our study has an important impact on clinical practice.

**Registration number:**

INPLASY202060025

## 1 Introduction

Parkinson disease (PD) is a common and progressive neurodegenerative disorder.^[1]^ PD affects over 6 million people worldwide in 2016 ^[1]^ and is the fastest growing in prevalence, disability, and deaths among the neurological disorders.^[2]^ Pathologically, PD is characterized by a loss of dopaminergic neurons in the substantia nigra and abnormal intracellular α-synuclein accumulation in the form of Lewy neurites and Lewy bodies in the brain.^[3]^ Clinically, PD is characterized by motor triad of resting tremor, bradykinesia, and rigidity and accompanying various nonmotor symptoms.^[4,5]^ However, the neurodegenerative process has started for years in the premotor phase before a diagnosis can be made.^[3,6]^ Sustained efforts have been made to develop reliable biomarkers for early detection, accurate diagnosis, and prognostic assessment.^[6]^

As a subunit of neurofilament, neurofilament light chain (NfL) is one of the major cytoskeletal components in mature neurons.^[7]^ Elevation of NfL concentrations in the cerebrospinal fluid (CSF) or blood is an index of neuronal/axonal injury and degeneration.^[7]^ While not disease-specific, NfL has been recognized as a promising diagnostic and prognostic biomarker in many neurological diseases, such as multiple sclerosis, Alzheimer disease, and amyotrophic lateral sclerosis.^[7–15]^ However, CSF and blood (ie, plasma and serum) NfL alterations in patients with PD from existing studies remain conflicting. Most of the studies reported increased NfL concentrations in the CSF and blood in patients with PD relative to healthy controls. ^[16–22]^ While some other studies found no significant difference in the CSF^[23–26]^ or blood^[25,27]^ NfL levels between patients with PD without dementia and healthy controls. To better understand these conflicting data, Wang et al conducted a meta-analysis that showed no significant difference in CSF NfL level between PD patients and controls.^[28]^ This result is interesting considering that PD is a neurodegenerative disease. However, it should be noted that their meta-analysis in PD included only 5 CSF studies. More recent studies that assessed NfL levels in the CSF and blood in PD have been published.^[19–22,25,27,29,30]^

In the present study, we will compile the recent evidence and perform 2 meta-analyses to quantitatively examine NfL levels in the CSF and blood separately in patients with PD compared to healthy controls.

## 2 Methods

### 2.1 Search strategies

We will comprehensively search PubMed, Embase, and Web of Science databases from each database's inception to 7th June, 2020 with no language or publication restrictions. The following search terms will be used: ((neurofilament light chain) OR nfl) AND ((Parkinson disease) OR Parkinson∗). Additional eligible studies will be obtained through cross-checking cited references. This protocol will conform to the Preferred Reporting Items for Systematic review and Meta-Analysis Protocols (PRISMA-P).^[31]^

### 2.2 Eligibility criteria

#### 2.2.1 Inclusion criteria

Studies will be included if they meet the following criteria:

(1) they were original and peer-reviewed articles published in English;
(2) they enrolled patients according to the established diagnostic criteria for idiopathic PD;^[5,32,33]^
(3) they were case-control studies that evaluated differences of NfL concentrations in the CSF or blood between idiopathic PD patients and healthy controls.

#### 2.2.2 Exclusion criteria

Publications will be excluded if:

(1) they lacked a healthy control comparison group;
(2) they lacked sufficient data to estimate the mean levels and standard deviation of CSF or blood NfL concentrations;
(3) they were nonhuman studies;
(4) the patient sample in one study was overlapped with those with a larger sample size in another study;
(5) they were not an original type, such as review, letter, case report, protocol, editorial, commentary, or conference abstract.
(6) In case of longitudinal studies, only baseline comparison results will be included.

**Figure 1:**
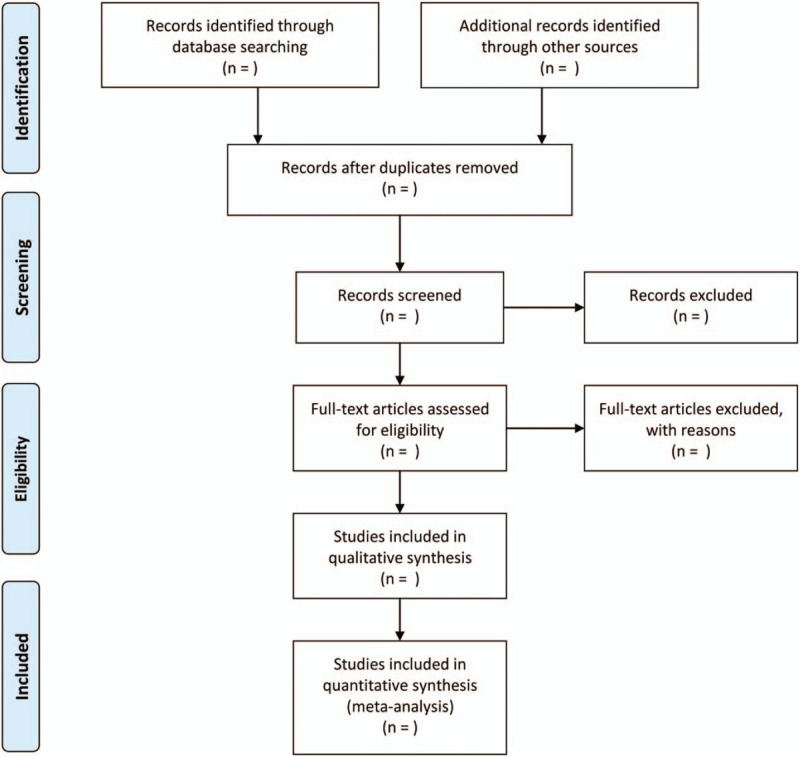
Study selection process following the preferred reporting items for systematic review and meta-analysis flowchart.

Figure 1 presents the process of selecting eligible articles according to the PRISMA statement.^[34]^ Literature search and study selection will be independently performed by 2 authors.

### 2.3 Data extraction

Data will be extracted from all eligible studies by two independent investigators using a standard form including the following information: The author's surname, year of publication, age, sex (male percentage), PD severity (Unified Parkinson Disease Rating Scale, part III score), H&Y stage, Mini-Mental State Examination (MMSE) score, disease duration (years), L-dopa equivalent daily dose (mg/day), and the mean and standard deviation of CSF NfL and blood NfL concentrations in PD patients and healthy controls, and NfL analysis methods.

### 2.4 Quality assessment

The quality of included studies will be assessed using the Newcastle Ottawa Scale (NOS).^[35]^ Using this tool, each study is judged on an 8-item scale with maximum score 9/9 categorized into dimensions (sample selection, comparability of groups, and the assessment of outcome). Quality assessment will be independently performed by 2 authors.

### 2.5 Data synthesis and statistics

Meta-analyses will be carried out using the STATA software version 13.0 (StataCorp, College Station, TX). Weighted and 95% confidence intervals for results of NfL concentration differences between the PD groups and the healthy control groups will be computed using a random-effects model. Heterogeneity will be assessed using the *I*^2^ statistic. Sensitivity analyses will be conducted to test the replicability of the results by repeating the same analyses by consecutively removing 1 study at a time. Potential publication bias will be assessed using Egger linear regression and funnel plot. Subgroup analyses will be conducted in patients with PD with and without dementia. Meta-regression analyses will be conducted to investigate whether NfL concentrations in the CSF and blood were confounded by the moderators, including age, gender, UPDRS-III, H&Y stage, L-dopa equivalent daily dose, and disease duration. The significance level will be set at *P* < .05.

### 2.6 Ethics and dissemination

This meta-analysis does not need Ethics approval because it will be performed using the data based on published studies. This meta-analysis will be published in a peer-reviewed scientific journal.

## 3 Discussion

There is an urgent need to develop a reliable diagnostic and prognostic biomarker in the management of PD. ^[6]^ In the last decade, a growing body of evidence supports the NfL as a biomarker of brain injury or neurodegeneration in CSF and blood in a variety of neurological disorders, which may have clinical promise.^[7]^ However, previous studies on NfL concentration in PD were inconsistent. The present meta-analysis will quantitatively examine whether NfL concentrations in the CSF and blood are elevated in patients with PD compared to healthy controls and examine what the magnitudes of their effect sizes are. This meta-analysis will provide the evidence of NfL concentrations in the CSF and blood in PD and we hope that our study has an important impact on clinical practice.

## Author contributions

**Conceptualization:** HongZhou Wang, LiJian Han, PingLei Pan

**Data curation:** HongZhou Wang, WanHua Wang

**Formal analysis:** HongZhou Wang

**Funding acquisition:** PingLei Pan

**Investigation:** HongZhou Wang, WanHua Wang, HaiCun Shi

**Methodology:** HongZhou Wang, HaiCun Shi

**Project administration:** LiJian Han, PingLei Pan

**Resources:** HongZhou Wang, WanHua Wang, HaiCun Shi

**Software:** HongZhou Wang

**Supervision:** LiJian Han

**Validation:** PingLei Pan

**Visualization:** HongZhou Wang

**Writing – original draft:** HongZhou Wang

**Writing – review & editing:** LiJian Han, PingLei Pan
